# 

**Published:** 1866-09

**Authors:** 


### CONTENTS.

### ORIGINAL CONTRIBUTIONS.

ART. XXX. Practical Remarks on Blood-Letting. [Concluded.] By D. B. Trimble, M.D., Chicago, Ill., ---- 513

ART. XXXI. Report of the Committee on Practical Medicine and Epidemics. By H. Noble, M.D., of Heyworth, Ill., Chairman, ---- 528

ART. XXXII. On the Cellular Vitality of the Tissues, as an Evidence of Life in New-Born Infants. By B. H. Cheney, M.D., of Joliet, Ill., ---- 538

ART. XXXIII. Abscess in the Testicle. By M. M. Eaton, M.D., of Peoria, Ill., ---- 549

ART. XXXIV. Irrigation of the Nasal Passages. By H. A. Johnson, M.D., of Chicago, ---- 552

### SELECTIONS.

On Cholera. By A. Clark, M.D., Professor of Pathology and Practical Medicine, College of Physicians and Surgeons, N.Y. ---- 553

### BOOK NOTICES.

Medical Electricity: embracing Electro-Physiology and Electricity as a Therapeutic, with Special Reference to Practical Medicine; Showing the Most Approved Apparatus, Methods, and Rules for the Medical Uses of Electricity in the Treatment of Nervous Diseases. By Alfred C. Garratt, M.D., Fellow of the Mass. Med. Society; Member of the Amer. Med. Asssociation, ---- 567

On Spermatorrhœa: Its Causes, Symptomatology, Pathology, Prognosis, Diagnosis, and Treatment. By Roberts Bartholow, A.M., M.D., Prof. of Physic and Med. Chemistry, in Med. College of Ohio; Lecturer on Clinical Medicine, and Physician to St. John's Hospital, Cincinnati, etc., etc., ---- 568

### EDITORIAL.

Chicago Medical Society, ---- 569

Encephaloid Tumor, ---- 569

Narcotism, ---- 569

Mortality for the Month of May, ---- 570

Mortality for the Month of July, ---- 571

Surgical Instruments, ---- 571

Obituary Record, ---- 571

Deodorizing Properties of Ground Coffee, ---- 572

